# Salvage surgery for advanced non-small cell lung cancer following previous immunotherapy: a retrospective study

**DOI:** 10.1186/s13019-023-02310-5

**Published:** 2023-07-20

**Authors:** Mitsunori Higuchi, Sho Inomata, Hikaru Yamaguchi, Takuro Saito, Hiroyuki Suzuki

**Affiliations:** 1 Department of Thoracic Surgery, Aizu Medical Center, Fukushima Medical University, 21-2 Maeda, Tanisawa, Kawahigashi, Aizuwakamatsu, 969-3492 Japan; 2 Department of Chest Surgery, Fukushima Medical University School of Medicine, Fukushima, Japan; 3 Department of Surgery, Aizu Medical Center, Fukushima Medical University, Aizuwakamatsu, Japan

**Keywords:** Non-small cell lung cancer (NSCLC), Salvage surgery, Immune checkpoint inhibitor (ICI), Downstaging, Prognosis

## Abstract

**Background:**

The development of systemic chemotherapy including immune checkpoint inhibitors (ICIs) has provided patients with unresectable advanced non-small cell lung cancer (NSCLC) an opportunity to undergo surgical intervention after initial treatment. However, no consensus regarding the indication for salvage surgery in these patients has been reached.

**Methods:**

We conducted a retrospective study of patients who underwent salvage surgery for advanced NSCLC (cStage IIIA–IVB) after treatment with ICIs from January 2018 to December 2022 at Aizu Medical Center and Fukushima Medical University Hospital. We evaluated the patients’ clinical data, calculated disease-free survival (DFS) and overall survival (OS), and assessed the survival benefit using the Kaplan–Meier method.

**Results:**

Thirteen patients underwent salvage surgery after immunotherapy. All patients achieved downstaging after initial chemotherapy. Eleven patients underwent lobectomy, and one patient underwent extirpation of intra-abdominal lymph nodes. The mean surgery time and intraoperative blood loss were 242.2 min and 415.1 g, respectively. The mean drainage period was 4.2 days (range, 2–9 days). Grade ≥ 3 postoperative complications were confirmed in three patients. The 2-year DFS rate was 71.2%, and the 2-year OS rate was 76.2%. A pathological complete response compatible with ypStage 0 was achieved in four (30.8%) patients. Patients with ypStage 0 and I achieved significantly better OS than those with ypStage ≥ II (*p *= 0.044), and patients without severe complications achieved significantly better DFS and OS than those with severe complications (*p *= 0.001 and *p *< 0.001, respectively).

**Conclusions:**

Salvage surgery after chemotherapy including ICIs is a feasible and effective treatment option for patients with advanced NSCLC, especially those who acquire downstaging to pathological stage 0 or I. However, severe perioperative complications might affect patient survival. A prospective study is urgently needed to evaluate the efficacy of salvage surgery.

## Background

Lung cancer has been a leading cause of death worldwide for several decades [1]. More than 60% of patients with lung cancer present with locally advanced or metastatic disease (Stage III to IV) at the time of diagnosis, at which point a surgical procedure may not be an option [2, 3].

Recent advances in chemotherapy including tyrosine kinase inhibitors (TKIs) and immune checkpoint inhibitors (ICIs) have contributed to the achievement of long-term cancer-free survival for patients with advanced non-small cell lung cancer (NSCLC) [4, 5]. However, some patients are found to have residual or relapsed lesions while continuing these chemotherapies. In such cases, the treatment options include irradiation, other chemotherapy, radiofrequency ablation, and other techniques. Salvage surgery is also a promising option for patients with advanced NSCLC. Several reports of salvage surgery for advanced NSCLC have described improved survival with acceptable surgical adverse events [4–6]. However, the early and long-term impacts of salvage surgery and the indications for such surgery remain unclear. In this study, we retrospectively analyzed patients with advanced NSCLC to elucidate their characteristics, perioperative complications, long-term survival with use of ICIs, and feasibility and efficacy of salvage surgery.

## Methods

### Study design and patient cohort

This study was approved by the institutional review board of Fukushima Medical University (IRB ID 2021–325), and we obtained individual patient consent. We conducted a retrospective review of patients with unresectable advanced NSCLC who were treated with immunotherapy from January 2018 to December 2022. During this period, we experienced 819 surgical cases for NSCLC. Thirteen patients (1.59%) had undergone salvage surgery after immunotherapy at Aizu Medical Center and Fukushima Medical University Hospital. The indications for salvage surgery in this study were achievement of significant downstaging from clinical stage IIIA to IVB at the time of diagnosis according to the eighth edition of the TNM staging system without progression after ICI therapy, reassessment for resectability, and maintenance of a good performance status between 0 and 1. Thoracic salvage surgery was defined as surgical intervention based on the standard operations (including lobectomy, pneumonectomy, wedge resection, and other procedures) for advanced NSCLC in patients who initially had no surgical indications but had achieved significant downstaging without progression after ICI therapy. The following demographic and clinical data were collected and reviewed from the electronic medical record library: age, sex, smoking history, histology, clinical stage, preoperative chemotherapy regimen, surgical procedure, surgery time, intraoperative blood loss, perioperative complications, chest drainage period, length of postoperative hospital stay, pathological diagnosis, pathological stage, postoperative therapy, and survival data.

### Statistical analysis

Data are described as mean and range for continuous variables and as percentage with 95% confidence interval for quantitative variables. Survival was assessed using the Kaplan–Meier method. The significance of the differences between the two groups was evaluated using the χ^2^-test and Fisher’s exact test. Statistical significance was defined as *p *< 0.05. Statistical analyses were performed using SPSS 28.0.1.0 (IBM Corp., Armonk, NY, USA).

## Results

### Patient characteristics

The patients’ clinicopathological characteristics are shown in Tables 1 and 2. The patients comprised eight men and five women with a mean age of 66.4 years (range, 52–83 years). All patients were diagnosed with NSCLC, including 10 patients with adenocarcinoma, 1 with squamous cell carcinoma, and 2 with large cell neuroendocrine carcinoma, staged from cStage IIIA to IVB at the time of diagnosis. Metastasis was found in the abdominal lymph nodes, pleura, lungs, bone, and brain. Maximum standardized uptake values (SUVmax) of 18F-fluorodeoxyglucose positron emission tomography (FDG-PET) before salvage surgery ranged from 0.0 to 12.7 (mean 6.15). Ten patients were negative for driver mutations, and one patient was positive for *BRAF* V600E mutation. The patients’ tumor proportion score (TPS) ranged from 1 to 95% (mean, 47.4%). The mean follow-up period after salvage surgery was 25.4 months.

**Table 1:** Clinicopathological characteristics before surgery (n = 13)

Age (y.o.)	66.4 (52–83)
Gender	Male: Female 8: 5
Brinkmann index	704.8 (0–1560)
Location	
Right upper lobe	7
Right middle lobe	0
Right lower lobe	3
Left upper lobe	2
Left lower lobe	1
Maximum tumor diameter (mm)	56.5 (14–96)
Histology	
Adenocarcinoma	10
Squamous cell carcinoma	1
Large cell neuroendocrine carcinoma (LCNEC)	2
c-stage	
c-stage IIIA	1
c-stage IIIB	5
c-stage IVA	5
c-stage IVB	2
Metastasis	Total number
Abdominal lymph nodes	1
Pleural dissemination	1
Lung	4
Bone	1
Brain	2
Driver mutation	
Negative	10
BRAF V600E	1
Not examined	2
Tumor proportion score (TPS)	
< 1%	0
1% ≤ < 50%	6
50% ≤	7
Preoperative chemotherapy	
Platinum, pemetrexed and pembrolizumab	6
Pembrolizumab	4
Platinum, VP16, and Atezolizumab	1
Platinum, pemetrexed, nivolumab, and ipilimumab	1
Nivolumab and ipilimumab	1
Tumor diameter before surgery (mm)	36.5 (8–110)
SUVmax before surgery	6.15 (0–12.7)
yc-stage	
yc-stage IA	3
yc-stage IB	3
yc-stage IIA	1
yc-stage IIB	1
yc-stage IIIA	4
yc-stage IVA	1
Time from initial treatment to surgery (months)	10.1 (2–40)

**Table 2:** Clinicopathological characteristics after surgery (n = 13)

Surgical procedure	
Lobectomy	8
Lobectomy with partial resection of another lobe	2
Lobectomy with partial resection of diaphragm	1
Lobectomy with reconstruction of pulmonary artery	1
Extirpation of abdominal lymph node	1
Ef	
Ef.0	2
Ef.1	2
Ef.2	5
Ef.3 (pathological complete response, pCR)	4
yp-stage	
yp-stage 0 (pCR)	4
yp-stage IA2	2
yp-stage IA3	1
yp-stage IIB	2
yp-stage IIIA	2
yp-stage IVA	2
Surgery time (minutes)	242.1 (139–446)
Intraoperative blood loss (g)	415.1 (25–2660)
Postoperative complications (grade)	Total number
Atelectasis (grade 1)	1
Atelectasis (grade 2)	2
Respiratory failure (grade 3)	1
Respiratory failure (grade 4)	2
Hemorrhage of gastric ulcer (grade 5)	1
Drainage period (median, days)	3.0 (2–9)
Postoperative hospital stay (median, days)	8.0 (7–150)
Total hospital stay (median, days)	12.0 (8–154)
Postoperative follow-up period (mean, months)	25.4 (2–51)
Total follow-up period (mean, months)	35.8 (9–63)
Postoperative chemotherapy	
Pemetrexed and pembrolizumab	2
Nivolumab and ipilimumab	1
Atezolizumab	1
None	9
Recurrence	3
Death	3

### Initial chemotherapy and preoperative condition

**Fig. 1:**
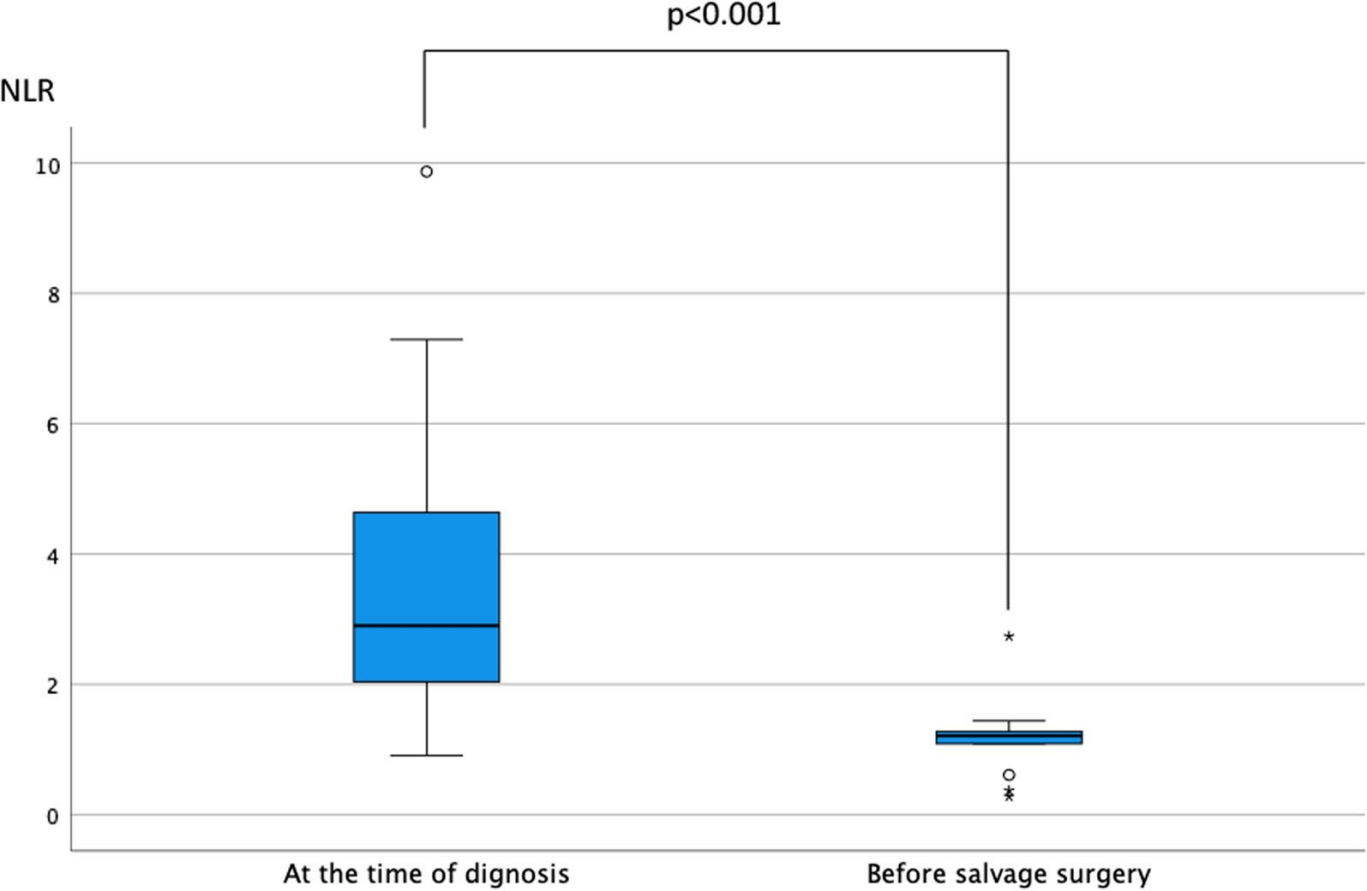
Comparison of NLR at the time of diagnosis and before salvage surgery. The mean NLR at the time of diagnosis and before salvage surgery was 3.75 (95% confidence interval, 2.60–5.16) and 1.16 (95% confidence interval, 0.88–1.50), respectively (*p *< 0.001). NLR, neutrophil-to-lymphocyte ratio

Preoperative chemotherapy included eight combination therapies (both cytotoxic agents and ICIs) and five ICI therapies. The mean duration of immunotherapy before surgery was 10.1 months (range, 2–40 months). The mean neutrophil-to-lymphocyte ratio (NLR) at the time of diagnosis and before surgery was 3.75 (range, 2.60–5.16) and 1.15 (range, 0.16–1.50), respectively (Fig. 1). The NLR decreased during immunotherapy, and the difference was statistically significant (*p *< 0.001). All cases except one had achieved downstaging (Table 1). Only one case had maintained same stage (cStage IIIA to ycStage IIIA).

### Salvage surgery and postoperative course

Lobectomy procedures with systemic lymph node dissection with or without adjacent organ dissection was performed in 12 patients, and extirpation of abdominal lymph nodes was performed in 1. All patients underwent open thoracotomy except one who underwent laparoscopic extirpation of abdominal lymph nodes. The mean surgery time and intraoperative blood loss were 242.2 min (range, 139–446 min) and 415.1 g (range, 25–2660 g), respectively. The median drainage period was 3.0 days (range, 2–9 days). Pathological findings revealed Ef.3 in four patients who achieved a pathological complete response (pCR). Maintenance chemotherapy after salvage surgery was performed in four patients according to the pathological staging (two received pemetrexed and pembrolizumab, one received nivolumab and ipilimumab, and one received atezolizumab). The other nine patients did not receive any chemotherapy with their consent.

### Adverse events and prognosis

**Fig. 2:**
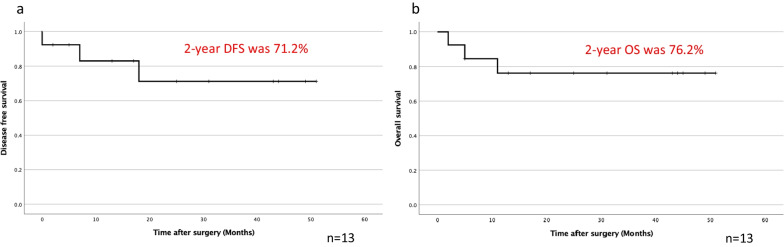
Postoperative **a** DFS and **b** OS after salvage surgery. The median DFS and median survival time were not reached, and the 2-year DFS and 2-year OS rates were 71.2% and 76.2%, respectively

**Fig. 3:**
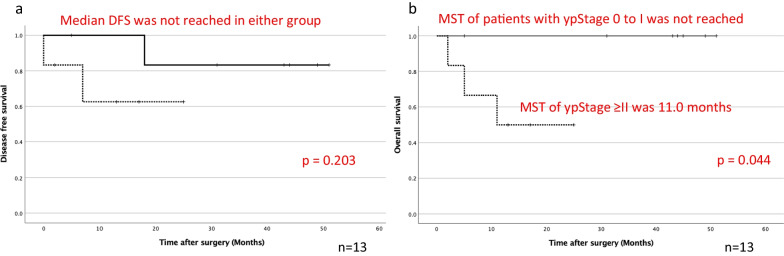
**a** DFS and **b** OS of patients with ypStage 0 to I (n = 7, solid line) and ypStage ≥ II (n = 6, dotted line). The median DFS was not reached in either group of patients (*p *= 0.203). The median survival time (MST) of patients with ypStage 0 to I was not reached, whereas that of patients with ypStage ≥ II was 11.0 months (*p *= 0.044). DFS, disease-free survival; OS, overall survival

**Fig. 4:**
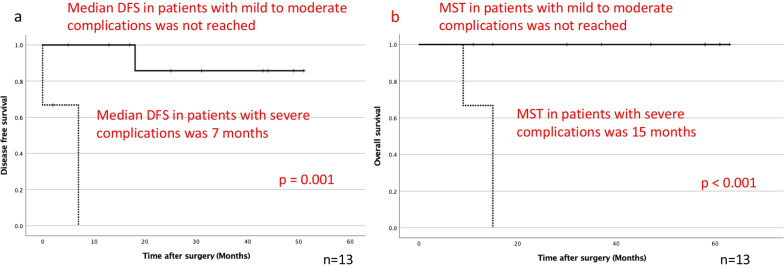
**a** DFS and **b** OS between patients with mild to moderate complications (n = 10, solid line) and severe complications (n = 3, dotted line). The median DFS in patients with mild to moderate complications was not reached, and that in patients with severe complications was 7 months (*p *= 0.001). The MST of patients with mild to moderate complications was not reached, whereas that of patients with severe complications was 15 months (*p *< 0.001). DFS, disease-free survival; OS, overall survival

Four (30.8%) of the 13 patients developed postoperative complications including atelectasis, respiratory failure, and hemorrhage of a gastric ulcer. The median postoperative hospital stay was 8.0 days (range, 7–150 days). There was one 90-day hospital death due to a hemorrhagic gastric ulcer with grade 4 respiratory failure. Three patients developed disease progression including tumor growth of the chest wall, mediastinal lymph node swelling, and multiple bone metastases. The 2-year disease-free survival (DFS) rate was 71.2%, and the median DFS was not reached (Fig. 2a). Three patients died due to progression of lung cancer, a hemorrhagic gastric ulcer, and melena, respectively. A case of hemorrhagic gastric ulcer was not due to immune-related adverse event, but postoperative physical stresses of surgery and mechanical ventilation, and a case of melena was due to immune-related adverse event. The 2-year overall survival (OS) rate was 76.2%, and the median survival time was not reached (Fig. 2b). The Kaplan–Meier curves exhibited a plateau in their tail. We also compared the prognosis stratified by postoperative pathological stage and postoperative high-grade complications. When we stratified the patients according to clinical stage after chemotherapy into a ycStage 0 to I group and ycStage ≥ II group, the DFS was not significantly different between the groups (*p *= 0.420), and the OS was also not significantly different (*p *= 0.091) (data not shown). Whereas when we also stratified the patients according to pathological stage into a ypStage 0 to I group and ypStage ≥ II group, the DFS was not significantly different between the groups (*p *= 0.203); however, the OS was significantly different (*p *= 0.044) (Fig. 3a, b). When we stratified the patients according to the presence of severe complications into a mild to moderate complication group and severe complication group, the DFS and OS were significantly different between the groups (*p *= 0.001 and *p *< 0.001, respectively) (Fig. 4a, b).

## Discussion

Bauman et al. [7] reported that the most common reasons for thoracic salvage surgery were obvious relapse as shown by computed tomography, persistently abnormal 18F-fluorodeoxyglucose positron emission tomography findings after completion of radiotherapy, and a delayed decision to convert to a trimodal approach. However, thoracic salvage surgery can be defined as surgical resection of residual or recurrent primary lung tumors after previous local or systemic treatments [8]. Several retrospective studies have shown the safety and feasibility of salvage surgery after initial treatment with TKIs or ICIs for advanced NSCLC [4, 5, 9]. Bott et al. [4] retrospectively examined nineteen patients who underwent lung resection after ICIs for metastatic or unresectable cancer including lung cancer. In that study, complications occurred in 32% of patients and the 2-year OS was 77%, which data were compatible with our results. Interestingly, Nakanishi et al. [10] reported effective local control and favorable survival outcomes by salvage surgery for patients with stage IIB to IIIB small cell lung cancer who received cisplatin, etoposide, and radiotherapy. Recent clinical trials of neoadjuvant treatment and adjuvant treatment for resectable NSCLC have shown that epidermal growth factor receptor-TKI and ICI therapy might provide a superior DFS benefit [11–14]. In the CheckMate 816 trial, Forde et al. [11] showed that neoadjuvant nivolumab plus chemotherapy resulted in significantly longer event-free survival and 24.0% of patients with a pCR by chemotherapy alone among patients with stage IB to IIIA resectable NSCLC. Our study showed four pCR cases (30.8%) which was compatible with the result of CheckMate 816 trial. In a phase-III trial (ADAURA trial) of patients with stage IB to IIIA epidermal growth factor receptor mutation-positive NSCLC, Wu et al. [12] found that DFS was significantly longer among those who received osimertinib than among those who received placebo. Additionally, the IMpower010 trial showed a DFS benefit with atezolizumab versus best supportive care after adjuvant chemotherapy in patients with resected stage II to IIIA NSCLC, with a pronounced benefit in patients whose tumors expressed programmed death ligand-1 (PD-L1) on ≥ 1% of tumor cells [13]. Furthermore, O’Brian et al. [14] performed an interim analysis of a randomized phase-III trial (KEYNOTE-091) and found that pembrolizumab significantly improved DFS compared with placebo and was not associated with new safety signals in completely resected, PD-L1-unselected, stage IB to IIIA NSCLC. These results might affect and improve the survival of patients with advanced NSCLC and even those with small cell lung cancer [10]. In the present study, the initial treatment regimen varied, including cytotoxic chemotherapy with an ICI, a single ICI, and double ICIs. The study also included patients during transition from separate treatment to combination treatment of cytotoxic agents and ICIs. We basically selected pembrolizumab as immunotherapy if the patient’s TPS was ≥ 50% and either nivolumab ± ipilimumab or atezolizumab if the TPS was < 50%. Essentially, we now choose combination therapy using both cytotoxic agents and ICIs except in patients aged > 80 years. These indications might differ among institutions.

Salvage surgery is generally considered technically more difficult with potentially higher morbidity than alternative treatments. Therefore, careful patient selection combined with surgical expertise can allow for successful salvage surgery with minimal morbidity [8]. Our data showed that OS was significantly better in patients with ypStage 0 to I cancer than in those with ypStage ≥ II cancer. Salvage surgery provides patients who were initially diagnosed with unresectable advanced NSCLC a chance of long-term survival, especially those who have achieved downstaging to ypStage 0 to I. These patients are probably good candidates for salvage surgery. However, ycStage should be more reliable information for making decisions of salvage surgery, although we could not show the results in this study. More precise preoperative staging is needed for the selection of candidates. Although we were able to safely perform salvage surgery, our study showed that the rate of grade ≥ 3 perioperative complications reached 23.1% and included respiratory failure and gastrointestinal hemorrhage. We also experienced one patient with a grade 5 complication (gastrointestinal hemorrhage), resulting in 90-day mortality, whose complications was due to postoperative physical stresses of both surgery and long-term mechanical ventilation. These severe perioperative complications might have affected the patients’ prognosis, and such patients might have lived longer if they had undergone continuation of chemotherapy rather than salvage surgery. In addition to these perioperative complications, the patients also developed many adverse events due to chemotherapy. Therefore, the candidates for salvage surgery should be selected with caution. However, we cannot estimate preoperatively which factors might contribute to perioperative severe complications. Therefore, we must carefully evaluate patients’ clinicopathological factors to select good candidates for salvage surgery in a multi-institutional prospective study. If severe postoperative complications related to the surgical procedure and immunotherapy occur, they should be managed as early as possible and the patients should be provided more intensive care.

We continued ICI treatment after salvage surgery except in patients with a pCR; however, the optimal time point at which to stop the ICI treatment was unknown. Recent reports including the IMpower 010 trial [13] and KEYNOTE-091 trial [14] showed that ICI treatment may be continued for around 1 year postoperatively as adjuvant chemotherapy. According to these reports, we could have stopped the ICI treatment at around 1 year. However, previous trials included only patients with pStage II to IIIA cancer. Our study included patients with more advanced cancer ranging from cStage IIIA to IVB. The difference in these populations might have affected the prognosis, and further prospective studies are therefore needed. Another serious issue that needs to be resolved is the lack of reliable biomarkers for predicting the effect and prognosis of immunotherapy. Several promising biomarkers are currently available, including PD-L1 expression, the tumor mutation burden, and gene expression signatures [15]. However, these factors are not reliable. After reviewing the clinical value of the NLR in patients with NSCLC treated with ICIs, Jiang et al. [16] set the NLR cutoff value at 5 and concluded that a high blood NLR (> 5) is associated with shorter progression-free survival and OS in patients treated with ICIs; they therefore suggested that the NLR has potential predictive and prognostic value. We also previously reported the usefulness of the NLR in a case report in which the patient, who was included in the present study, achieved a pCR after immunotherapy [17]. In the present study, the NLR of each patient decreased after immunotherapy and persistently remained between 1 and 2 during immunotherapy (Fig. 1). The NLR is simple to measure and might serve as a reliable biomarker of immunotherapy.

Our study has some limitations, including its retrospective nature, small number of highly selected patients after immunotherapy with better outcomes, better performance score and not multiple metastases, and also including heterogeneous population (e.g., cStage IIIA–IVB, various histologic types, and various immunotherapy regimens). Furthermore, pCR in some patients does not mean that the metastases in other places are also completely gone, so pCR should be noted only occurred within intrapulmonary lesions and lymph nodes, although we could evaluate the condition with image devices such as CT scan and FDG-PET. A prospective study is needed to evaluate the safety and feasibility of salvage surgery for patients with initially unresectable lung cancer. We should also compare the prognosis of patients with advanced lung cancer who undergo salvage surgery versus those who undergo multidisciplinary treatment without salvage surgery. Such studies will show the real efficacy of salvage surgery.

Local treatment such as salvage surgery in addition to systemic chemotherapy has an important role in the control of advanced lung cancer. Prospective studies of salvage surgery after ICI therapy are urgently needed to improve the prognosis of initially unresectable advanced NSCLC.

## Conclusions

Salvage surgery after chemotherapy, such as ICI therapy, is a feasible and effective treatment option for patients with advanced NSCLC, especially those who have achieved downstaging to ypStage 0 to I. However, severe perioperative complications might affect patient survival. A prospective study is urgently needed to evaluate the efficacy of salvage surgery.

## Data Availability

Data will not be shared because the patients’ privacy is protected.

## Abbreviations

DFS: Disease-free survival
FDG-PET: 18F-fluorodeoxyglucose positron emission tomography
ICI: Immune checkpoint inhibitor
MPR: Major pathological response
MST: Median survival time
NLR: Neutrophil-to-lymphocyte ratio
NSCLC: Non-small cell lung cancer
OS: Overall survival
pCR: Pathological complete response
PD-L1: Programmed death ligand-1
SUVmax: Maximum standardized uptake value
TKI: Tyrosine kinase inhibitor
TPS: Tumor proportion score
